# The role of systematic errors

**DOI:** 10.1093/nsr/nwab082

**Published:** 2021-05-22

**Authors:** Antonio Navarra

General circulation models (GCMs) have progressed enormously over the last few decades and they have allowed huge advances in forecasting at every timescale, from daily to seasonal and decadal, and in climate simulations of the Earth climate system. These advances have increased the relevance of the results for decision-making and the drafting of strategies and policies with far-reaching impacts. In fact, they provide the foundation of the global conversation on climate change mitigation and adaptation. Even the complex international negotiation, taking place in the context of the Paris Agreement and the UN Framework Convention on Climate Change, is ultimately based on science obtained mostly with global and regional models.

It is not surprising then that the issues of the accuracy, fidelity and reproducibility of these results are of foremost importance. Deviation from reality in models occurs either as a random effect, i.e. different from simulation to simulation as a result of the sensitivity of the system nonlinear interactions to perturbations, or as a systematic deviation, showing in every simulation and usually typical of a certain model. The latter is also known as ‘systematic error’ and sometimes ‘bias’. The systematic error can present itself as a deviation of the mean or as a systematic misrepresentation of some of the statistics of the system, for instance as an over- or underestimation of frequency and intensity of particular events.

Certain errors are particularly stubborn. The double Intertropical Convergence Zone (ITCZ) has plagued GCMs for a long time and it is resistant to improvements in resolution and formulation [1,2] and in the Atlantic has affected forecasting skills [3,4]. The systematic error is probably just a symptom of various inaccuracies and/or errors that ultimately show in a specific form, but they can affect not only at the local level but also through remote teleconnection. For instance, the double ITCZ can affect climate variability outside the tropics [5]. GCMs provide also the basic information for all the studies aiming at localizing information, for instance as boundary condition in regional models, and in this way the systematic error can propagate into downscaling exercises [6]. It is increasingly evident how the influence of this error can be pervasive through the entire climate enterprise.

It seems reasonable that a good understanding of the causes and effects of systematic errors is of utmost importance for weather and climate investigations, especially if the assessment has to become the basis for policy formulation or implementation measures. It simply cannot be ignored and it is crucial to define the limits and content of our knowledge. In this issue, Tang *et al.* [7] provide a nice example of an investigation that takes systematic error into consideration for a problem that is particularly relevant to planning for adaptation to climate change and to a correct evaluation of the connected risk. ENSO variability has large impacts at seasonal scale in many areas of the world and understanding its evolution under global change is central to the definition of the adaptation strategies.

ENSO is identified usually as a deviation from the climatology, i.e. a long-term mean state, but climate change will act on both the mean and the variability. So, it is an issue if criteria based on present-day thresholds can still be used in climate projections and what is the best strategy to modify them to give an accurate representation of ENSO in a changing climate. The systematic error has proven to be resistant and chances are that it is not going to go away any time soon. Barring sudden breakthroughs, we will have to find ways to go around it and to assess correctly its impacts on processes and on the predictive skill. The most promising opportunities to deal with systematic errors are offered by the rapid developments in recursive data exploration, machine learning and powerful nonlinear analysis techniques that are currently underway. The combination of advanced GCMs and sophisticated data exploration will probably give us the best opportunity to tackle this stubborn problem.

***Conflict of interest statement*.** None declared.
